# Sex-Dependent Phenotypic and Histomorphometric Biomarkers in the APPswe/PS1dE9/Blg Mouse Model of Alzheimer’s Disease

**DOI:** 10.3390/brainsci15111237

**Published:** 2025-11-18

**Authors:** Elena Kuzubova, Alexandra Radchenko, Mikhail Pokrovskii, Olesya Shcheblykina, Kirill Chaprov, Arkadii Nesterov, Tatiana Avtina, Vladimir Pokrovskii, Mikhail Korokin

**Affiliations:** 1 Research Institute of Pharmacology of Living Systems, Belgorod State National Research University, 85 Pobedy St., Belgorod 308015, Russia; radchenko_a@bsuedu.ru (A.R.); pokrovskii@bsuedu.ru (M.P.); shcheblykina@bsuedu.ru (O.S.); nesterov_a@bsuedu.ru (A.N.); avtina_t@bsuedu.ru (T.A.); pokrovskiy@bsuedu.ru (V.P.); korokin@bsuedu.ru (M.K.); 2 Institute of Physiologically Active Compounds at Federal Research Center of Problems of Chemical Physics and Medicinal Chemistry, Russian Academy of Sciences, Chernogolovka 119071, Russia; chaprov@ipac.ac.ru

**Keywords:** dementia, Alzheimer’s disease, amyloid plaques, behavior, sexual dimorphism

## Abstract

**Background**: Sex-related differences significantly impact biomedical research outcomes, yet female subjects are often excluded due to concerns about variability from the estrous cycle. This study aimed to investigate the sex-dependent differences in behavioral phenotypes and amyloid-beta plaque accumulation in the APPswe/PS1dE9/Blg transgenic mouse model of Alzheimer’s disease. **Methods**: Male and female APPswe/PS1dE9/Blg transgenic mice and wild-type (WT) controls were assessed at 7.5 and 10 months of age. A comprehensive behavioral test battery was employed, including the Open Field, Novel Object Recognition, Y-Maze, and Barnes Maze tests. Histological analysis of amyloid plaque was carried out. **Results**: Female transgenic mice displayed delayed accumulation of Aβ plaques and milder cognitive decline compared with males. At 10 months, plaque load in females corresponded to that of 7.5-month-old males, demonstrating a temporal lag in pathology. Behavioral impairments correlated negatively with cortical plaque burden (r = −0.4964, *p* = 0.0181), supporting its role as a structural biomarker of disease progression. **Conclusions**: This study identifies distinct sex-dependent trajectories of behavioral and histomorphometric biomarkers in APPswe/PS1dE9/Blg mice. Females exhibit delayed amyloid pathology and cognitive decline, suggesting intrinsic neuroprotective mechanisms that modulate biomarker expression over time. These findings emphasize the necessity of integrating both sexes in preclinical biomarker research and support the use of morphometric endpoints as translationally relevant indicators of Alzheimer’s disease progression.

## 1. Introduction

In biomedical research, the choice of age, sex, and number of subjects per group is often a critical question. These aspects become even more sensitive in studies involving transgenic animals [1,2,3].

Researchers frequently neglect female subjects in their studies due to the risk that the hormonal cycle may reduce the homogeneity of study groups and distort the results of experimental manipulations [4,5]. It was long believed that due to the estrous cycle, female rodents exhibit greater natural variability than males. However, it is this very belief that has perhaps become the main barrier in combating gender bias in preclinical research [6].

Indeed, differences exist between the sexes at the genetic, cellular, and biochemical levels; some of these are endogenous, while others have an epigenetic origin, and many are unrelated to sex hormones and are commonplace [7,8]. Females are characterized by cyclic changes in the levels of sex hormones (estrogens and progesterone), which affect metabolism and behavior. Males have a more stable hormonal background, with a dominant level of testosterone [9].

Blood analyses also reveal differences: females have an albumin level almost 1.5 times higher than males, and an increased albumin-globulin ratio (by 60%). Males show elevated concentrations of alkaline phosphatase and urea. The percentage of lymphocytes in the blood of females is consistently higher compared to that in males. Males exhibit greater variability in the relative monocyte count (coefficient of variation over 37%) [10,11,12,13].

The gut microbiome of mice also has its specificities. Males have lower microbial diversity and density of the bacterial population. For example, B6 females show a predominance of Lactobacillus plantarum and Bacteroides distasonis, while BALB/c females exhibited a dominance of bifidobacteria [14,15,16].

Females typically demonstrate a more active immune response, making them more resistant to infections but simultaneously more susceptible to autoimmune diseases. For instance, upon immune stimulation, Th2 cells are primarily activated in females, whereas Th1 cells are activated in males [17,18,19]. Macrophages in males tend towards M1-type activation, while in females they tend towards M2-type [20].

It is also known that sexual dimorphism strongly influences various behavioral patterns in individuals (social behavior, anxiety states). For example, female rodents are generally less aggressive than males, not only towards each other but also towards human experimenters [21]. Pain threshold, the frequency of chronic pain, and the intensity of pain sensations also depend on sex [22]. Thus, for instance, Cahill argued that sex can alter, negate, or even reverse research results; and that the phenotypic effects of a gene knockout in one sex may be entirely absent or even opposite in the other sex with the same knockout [23].

In turn, Mogil and Chanda conducted studies on 40 mouse lines across 3 different laboratories and showed that females, tested at random points of their estrous cycles, were no more variable than males, and that estrous cyclicity either does not affect variability, or male mice possess their own sex-dependent variability [24].

In the context of Alzheimer’s disease, biomarkers play a central role in both diagnosis and translational modeling. Current research distinguishes between molecular (Aβ, tau, neurofilament light chain), neuroimaging (PET, MRI), and histomorphometric biomarkers that reflect disease progression at the tissue level. The APPswe/PS1dE9/Blg (APP/PS1) model is widely used to evaluate such structural biomarkers, as amyloid plaque density and distribution that directly correlate with cognitive impairment and neurodegeneration severity [25,26]. Despite the extensive characterization of this model, sex-dependent variability in biomarker expression remains insufficiently understood [27,28].

Therefore, this study aimed to characterize sex-dependent phenotypic and histomorphometric biomarkers in the APP/PS1 mouse model of Alzheimer’s disease.

## 2. Materials and Methods

### 2.1. The Laboratory Animals

The study was conducted on mice of the APP/PS1 subline. To minimize the subjective assessment of the results of the study, blind data analysis was applied, in which the researchers did not have information about the belonging of samples to specific groups until the completion of the main phase of the analysis [29,30]. Randomization was carried out depending on body weight before the start of the experiment. The groups were formed as follows: control group WT, females, n = 15; group WT, males, n = 15; group APP/PS1, females, n = 15; and group APP/PS1, males, n = 15, aged 7.5 and 10 months. Female mice were tested regardless of the cycle phase [31]. A new cohort of mice was taken for each time point. The control group was formed from the same litter as the experimental one.

The animals were housed in the SPF vivarium of Belgorod State National Research University under standard conditions, including a standard diet, ad libitum access to food and water, a 12-h light/dark cycle, an ambient temperature of +22 to +24 °C, and a relative humidity of 50–65%.

Our laboratory systematically monitored possible biases in behavioral tests. The day after the last behavioral test, the mice were sacrificed, and their brains were extracted for histological analysis of amyloid plaque formation using Congo red staining.

Mice were terminally anesthetized and sacrificed by CO_2_ and isoflurane inhalation for subsequent material collection (RWD Life Science Co., Shenzhen, China).

### 2.2. Genotyping of Transgenic Animals

Genotyping was performed using primers proposed by Jankowsky et al. [32,33], which amplify a region of the transgene cassette (if present) and a region of the mouse genomic DNA simultaneously.

The reaction utilized a mixture of three primers: a common reverse primer, PrP_rev (5′-GTG GAT ACC CCC TCC CCC AGC CTA GAC C-3′), homologous to the sequence of the PrP gene in the mouse genome and within the transgene cassette; a forward primer, PrP_for (5′-CCT CTT TGT GAC TAT GTG GAC TGA TGT CGG-3′), homologous to a region of the genomic mouse PrP that was removed from the MoPrP.Xho vector; and specific forward primers for PS1 (5′-CAG GTG GTG GAG CAA GAT G-3′) and APP (5′-CCG AGA TCT CTG AAG TGA AGA TGG ATG-3′), homologous to the sequences of the mutant proteins within the transgene cassette. The amplification protocol was as follows: 1 cycle—95 °C for 3 min; 30 cycles—95 °C for 20 s, 55 °C (for APP) or 65 °C (for PS1) for 20 s, 72 °C for 20 s; and 1 cycle—72 °C for 2 min.

### 2.3. Histological Studies

**Figure 1:**
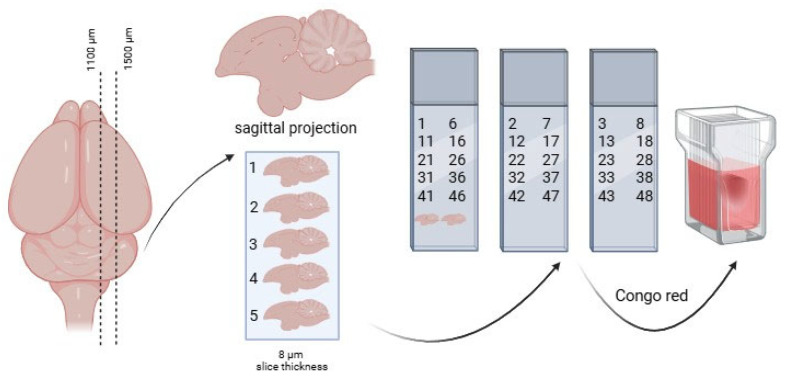
Layout scheme of brain sections during the preparation of histological specimens. From a 400 µm thick brain region, 5 slides were prepared, each consisting of 10 brain sections; every fifth brain section was placed on each slide.

After euthanizing the animals with a narcotic overdose at the ages of 7.5 and 10 months, the brains were extracted and fixed in Carnoy’s solution (96% ethanol, chloroform, glacial acetic acid in a 6:3:1 ratio) [34,35,36]. Subsequently, histological sections with a thickness of 7–8 µm were prepared using a standard protocol and stained with Congo red [37,38]. The scheme for organizing the section groups for the morphometric analysis of inclusions is presented in Figure 1.

Microscopy of the specimens was performed using a Nikon Eclipse Ti (Amstelveen, The Netherlands) microscope equipped with a motorized stage. Panoramic imaging of the mouse brain sections in TRITC fluorescence mode was conducted with a 10× objective using the NIS Elements AR software (version 4.6, Laboratory Imaging, Prague, Czech Republic), with frame stitching into a single composite image, 10% overlap, and automatic post-processing.

The resulting images were uploaded into QuPath software (version 0.5.1, Belfast, Northern Ireland, UK) for the detection and analysis of aggregates. Object detection was based on the brightness threshold of amyloid fluorescence spots relative to the baseline brightness of spots in intact brain tissue (Threshold from 90 to 100; smoothing sigma 1). If an incorrect selection was detected, the object was manually deleted.

Morphometric data was expressed in % plaque area/mm^2^ cortex. Following automatic object detection, a manual review was conducted to exclude incorrect identifications. To confirm the reproducibility of the results, calculations were carried out by two independent researchers [39,40]. The data between the 2 counts differed by less than 5%.

### 2.4. Behavioral Testing

#### 2.4.1. Open Field Test

The test animal was placed in the Open Field (OpenScience, Krasnogorsk, Russia) apparatus, and its movements were recorded. The apparatus is a square chamber with a 50 × 50 cm base, made of opaque plexiglass. The animal’s behavior was assessed based on the parameters characterizing locomotor activity in mice. The software automatically provided the selected parameters for analysis: distance traveled, activity, and average speed of all movements (cm/s). Each animal was tested for 5 min under standard room lighting [41,42,43].

#### 2.4.2. Novel Object Recognition Test

This is a simple behavioral test based on the innate exploratory behavior of rodents. The test is divided into three phases: habituation, training/adaptation, and the testing phase. On the first day of the test, the animal is placed in an empty 50 × 50 cm arena (OpenScience, Krasnogorsk, Russia) to explore for 5 min under standard room lighting. The second day is the adaptation phase, when the animal is placed in the same arena with two identical objects. On the third day, the testing phase, the animal is placed in the arena with one familiar object from the previous phase and one novel object. The following parameters were recorded: the number of approaches to the novel and familiar objects and the time spent near each [44,45,46].

#### 2.4.3. Y-Maze

Working memory was assessed using a Y-maze (OpenScience, Krasnogorsk, Russia) with arm dimensions of 32.5 × 8.5 × 15 cm (L × W × H.) The test was conducted under standard room lighting (40 lux). Mice were first allowed to explore two arms of the maze for 5 min, while the third arm was blocked. After a 30-min inter-trial interval, a second trial was conducted, during which the mice were allowed to explore all three arms for 5 min total. An entry into an arm was recorded when more than half of the mouse’s body crossed the boundary between two arms. The number of entries and the time spent in each arm were recorded. Analysis was performed using two scenarios: the entire 5-min test duration, or the first 2 min of “active exploration” [47,48].

#### 2.4.4. Barnes Maze Test

This test is used to study spatial learning and memory in animals. The goal of the Barnes Maze (OpenScience, Krasnogorsk, Russia) is for the mouse to explore the space and remember the location of the escape hole using the configuration of distal visual cues placed around the testing area. The setup is a circular platform 122 cm in diameter, containing 40 holes, each 5 cm in diameter, one of which serves as the exit (escape box). The distal visual cues consisted of four black-and-white images with different shapes and patterns, positioned in the north, south, west, and east directions. Video recording was performed for 5 min. Measurements included the total distance traveled by the animal, movement speed, and finding the exit within the allotted time [49,50].

### 2.5. Statistical Analysis

All the obtained results of behavioral activity were subjected to statistical processing using GraphPad Prism 8.0 software (San Diego, CA, USA); a two-way ANOVA followed by Tukey’s post hoc test was also used. The normality of the distribution was checked using the Kruskall-Wallis test (* *p* < 0.05; ** *p* < 0.01; *** *p* < 0.001; ns) and Gehan-Breslow-Wilcoxon test. No datapoints were excluded from analysis. The significance level was set at 95% (*p* < 0.05). For each test, 2 independent comparisons were carried out, the dependence of the results on the genotype and the dependence of the test results on gender (all data are shown in the figures below). Correlation analysis of the dependence of amyloid accumulation on behavioral testing was performed using Pearson’s chi-square test.

## 3. Results

Analysis of locomotor and exploratory activity parameters in the Open Field test in female and male APP/Ps1 and wild-type (WT) mice at 7.5 months of age did not reveal any statistically significant intergroup differences (*p* > 0.05). As the data demonstrate, the average movement speed (cm/s) was comparable in animals of all experimental groups. Similarly, the total distance traveled showed no significant differences between groups, indicating no effect of genotype on basal locomotor activity.

**Figure 2:**
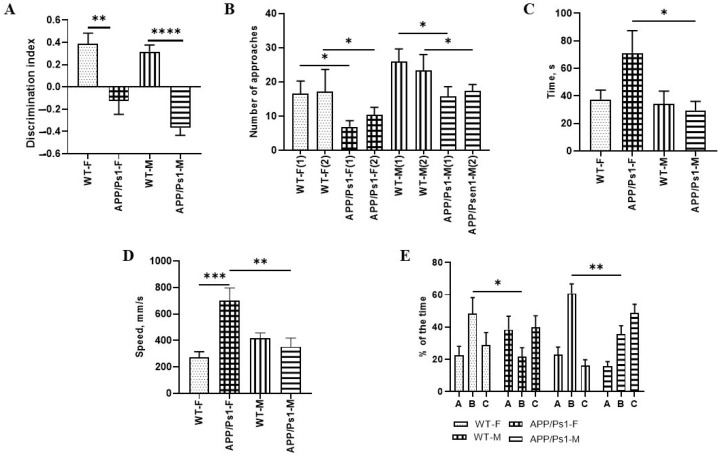
Analysis of behavioral testing in males and females of the APP/Ps1 line at the age of 7.5 months. (**A**,**B**) Indicators of preference for a “new” toy in female and male mice in a test for recognizing new objects. (**C**) Parameters of anxiety behavior in females and males of the APP/Ps1 line and controls in the Open Field test. (**D**) Mouse activity indicators in the Y-Maze Test. (**E**) The preference between the “old—**C**” and “new—**B**” arm in the Y-Maze Test. * *p* < 0.05; ** *p* < 0.01; *** *p* < 0.0004; **** *p* < 0.0001.

Likewise, the number of entries into the central zone of the arena did not differ between WT and APP/Ps1 animals, regardless of sex. However, the time spent in the center was significantly higher in APP/Ps1 females compared to males of the same line (*p* = 0.033, Kruskall-Wallis test, Figure 2C), which may indicate sex-dependent differences in anxiety-like behavior.

Quantitative analysis of locomotor activity parameters (distance traveled and average speed of movement) over the 5-min testing period revealed no statistically significant differences between the experimental groups. The obtained data indicate a comparable level of basal motor activity in all study animals, confirming the homogeneity of the experimental groups for this parameter.

Wild-type (WT) mice demonstrate a significantly greater number of approaches to the novel object compared to APP/Ps1 transgenic animals, with this difference observed in both sexes. Although APP/Ps1 mice showed some increase in exploratory activity by the second testing session, their performance remained significantly lower than that of WT animals. The differences in females between the control group and the experimental group on the first day are lower compared to those on the second day (*p* = 0.41, *p* = 0.16); in males we can observe a different picture (F (7, 100) = 2620, *p* = 0.24, *p* = 0.44, Sidak’s multiple comparisons test, Figure 2B).

The Discrimination Index (DI) reflects the ability to discriminate between familiar and novel objects. In WT females, DI was 0.38 ± 0.05 (*p* = 0.0056, Kruskall-Wallis test, Figure 2A), indicating a preference for the novel object, whereas in APP/Ps1 females, DI was negative −0.13 ± 0.08, indicating impaired recognition.

Intergroup differences were also identified in males: WT males demonstrated a positive DI (0.32 ± 0.06), whereas APP/Ps1 males had an RI below zero (−0.36 ± 0.04, *p* < 0.0001, Kruskall-Wallis test, Figure 2A), confirming a deficit in novel object recognition.

Analysis of the results from the Y-maze test revealed no statistically significant differences in the distance traveled between males and females (*p* > 0.05). However, significant genotype-dependent differences in movement speed were found. Specifically, APP/Ps1 females demonstrated a significantly higher speed compared to WT females (*p* = 0.0001), which may indicate hyperactivity. Interestingly, APP/Ps1 females also exhibited a higher speed than WT males (F (3, 41) = 8.684, *p* = 0.0181, one-way ANOVA, Figure 2D). These data confirm the presence of pronounced sexual dimorphism in the behavioral manifestations of APP/Ps1 transgenic model animals, where females show signs of hyperactivity, while males exhibit reduced locomotor activity compared to that in controls.

The data indicate that WT females show a strong trend towards spending more time in the novel (B) arm compared to APP/Ps1 females (*p* = 0.06). Among males, a similar trend is observed, with WT males spending more time in the novel (B) arm than APP/Ps1 males (*p* = 0.055). However, the percentage of time spent in the novel arm did not differ between APP/Ps1 males and females (*p* = 0.5214). No significant differences were found between WT females and males either (F (6, 195) = 8.480, *p* = 0.2783 one-way ANOVA, Figure 2E).

**Figure 3:**
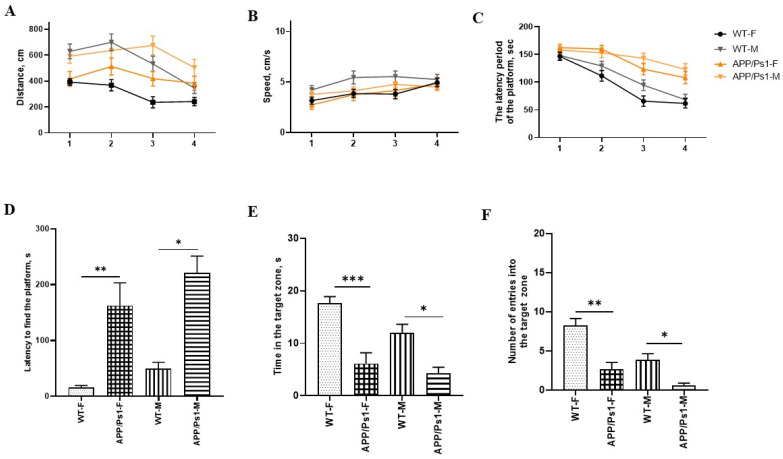
The Barnes Maze test at the age of 7.5 months. (**A**) Distance, and (**B**) speed during all 4 days of training. (**C**) The time spent searching for “shelter” is the average per day for 4 attempts. (**D**) The latent time spent in the “shelter” on the test day, and (**E**) the time spent in this zone for a total of 5 min of the attempt. (**F**) The number of returns to the “shelter” area within 5 min. * *p* < 0.05; ** *p* < 0.01; *** *p* < 0.0004.

As can be seen from the data, there were no statistical differences in distance and speed between females and males during all 4 days (Figure 3A,B). In male APP/Ps1 mice, the latency to find the platform showed no statistically significant improvement over the training period, suggesting a lack of learning progression.

In contrast, female APP/Ps1 mice exhibited a progressive decrease in escape latency with each successive training day, demonstrating a typical learning curve. However, the control (WT) female group achieved significantly shorter latencies. Thus, the difference between the females from the control group and the experimental group is statistically different from the 2nd to the 4th day of training (F (9, 564) = 2.443, day 2—*p* = 0.0006, day 3—*p* = 0.0003, day 4—*p* = 0.0061, Tukey’s multiple comparisons test, Figure 3C). Males have a similar pattern (training (F (9, 564) = 2.443, day 2—*p* = 0.0289, day 3—*p* = 0.0022, day 4—*p* = 0.0012, Tukey’s multiple comparisons test, Figure 3C). Based on these findings, it can be concluded that at 7.5 months of age, female APP/Ps1 mice retain a functional learning capacity, but its efficiency is compromised compared to healthy controls, likely due to emerging pathological changes.

The behavioral profile of the animals on the test day (at 7.5 months of age) in the Barnes Maze revealed significant differences between groups in the latency to find the platform, time spent in the target zone, and the number of entries into the target zone.

The latency to find the platform was significantly increased in APP/Ps1 females compared to that in WT females (*p* < 0.01, Kruskall-Wallis test, Figure 3D), as well as in APP/Ps1 males compared to WT males (*p* < 0.05, Figure 3D). The time spent in the target zone was significantly reduced in APP/Ps1 females compared to that in the control group (*p* < 0.001, Kruskall-Wallis test, Figure 3E) and was also decreased in APP/Ps1 males relative to that in controls (*p* < 0.05, Kruskall-Wallis test, Figure 3E), indicating impaired long-term memory. The number of entries into the target zone was substantially higher in the control group compared to those in both APP/Ps1 females (*p* < 0.01, Kruskall-Wallis test, Figure 3F) and APP/Ps1 males (*p* < 0.05, Kruskall-Wallis test, Figure 3F), suggesting more active search behavior in non-transgenic animals.

Sex-dependent differences in this test were identified during the training days and were associated with the rate of learning, but were absent on the test day during the assessment of long-term memory.

**Figure 4:**
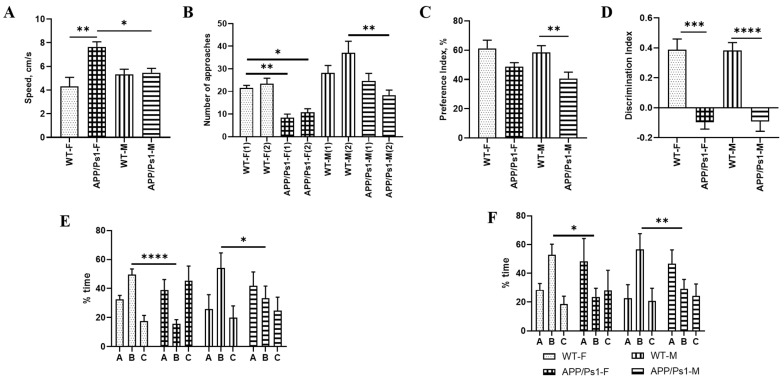
Analysis of behavioral testing in males and females of the APP/Ps1 line at the age of 10 months. (**A**) Activity indicators in the New Object Recognition test. (**B**) Indicators of preference for a “new” toy in female and male mice in a test for recognizing new objects. (**C**) Preference index in the New Object Recognition test, and (**D**) discrimination index in the New Object Recognition test. (**E**) The preference between the “old—**C** ” and “new—**B**” arm in the Y-Maze Test within 2 min, and (**F**) The preference between the “old—**C** ” and “new—**B**” arm in the Y-Maze Test within 5 min. * *p* < 0.05; ** *p* < 0.01; *** *p* < 0.0004; **** *p* < 0.0001.

As the test results show, locomotor function and general psychomotor-emotional status remain unchanged between female and male groups over time (from 7.5 to 10 months). APP/Ps1 mice demonstrate increased activity compared to WT mice at both 7.5 and 10 months of age (F (3, 89) = 3.697, *p* = 0.0083, *p* = 0.0499, one-way ANOVA, Figure 4A). Notably, at 7.5 months, female APP/Ps1 mice spend significantly more time in the center of the arena, reflecting either reduced anxiety-like behavior or altered exploratory behavior at a younger age. By 10 months, these behavioral differences become less pronounced, primarily manifesting as a generally increased activity in APP/Ps1 mice, though with high variability.

There were no differences in locomotor activity between mice aged 7.5 and 10 months. Male and female APP/Ps1 mice at the age of 10 months showed less interest in the new object compared to wild-type mice (F (2809, 44.95) = 6190, 1 day (F)—*p* = 0.0044, 2 day (F)—*p* = 0.0353, 1 day (M)—*p* = 0.0043, one-way ANOVA, Figure 4B).

With age (from 7.5 to 10 months), the preference index in APP/Ps1 males decreases (*p* = 0.0072, Kruskall-Wallis test, Figure 4C), and the discrimination index significantly worsens in both male and female APP/Ps1 mice compared to WT (*p* = 0.0006, *p* < 0.0001, Kruskall-Wallis test, Figure 4D). This indicates a progressive deterioration of long-term memory, specifically the ability to recognize a novel object in APP/Ps1 mice, along with a decline in their interest in the novel object, particularly in males. In WT mice, the indices remain stable in both females and males.

After 2.5 months, activity levels in the groups remain unchanged. Short-term memory impairments in transgenic animals also remain pronounced over time. The differences between healthy control and experimental groups, depending on gender, remain, both for the first 2 min (F (2, 36) = 1.733, *p* = 0.0346, *p* = 0.0004, one-way ANOVA, Figure 4E) and for the entire test period (F (6, 90) = 2.210, one-way ANOVA, *p* = 0.0036 (M), *p* = 0.0241(F), one-way ANOVA, Figure 4F).

**Figure 5:**
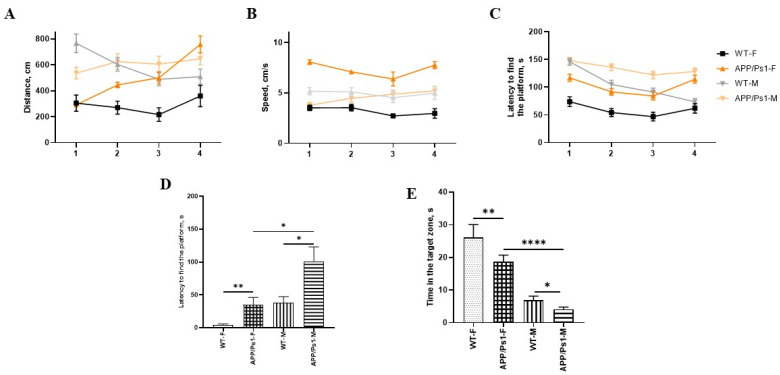
The Barnes Maze test at the age of 10 months. (**A**) Distance, and (**B**) speed during all 4 days of training. (**C**) The time spent searching for “shelter” is the average per day for 4 attempts. (**D**) The latent time spent in the “shelter” on the test day, and (**E**) the time spent in this zone for a total of 5 min of the attempt. * *p* < 0.05; ** *p* < 0.01; **** *p* < 0.0001.

After 2.5 months, female APP/Ps1 mice showed increased activity compared to the control group, both in speed (F (9, 186) = 3.378, 1 day—*p* < 0.0001, 2 day—*p* < 0.0001, 3 day—*p* < 0.0001, 4 day—*p* < 0.0001, Tukey’s multiple comparisons test, Figure 5B) and distance traveled (F (9, 213) = 8.697, 1 day—*p* = 0.0191, 2 day—*p* = 0.0020, 3 day—*p* = 0.0039, Tukey’s multiple comparisons test, Figure 5A). Their learning ability, measured by the reduction in latency to find the platform, is impaired. In contrast to their performance at 7.5 months, female transgenic mice show no learning progression across days, indicating marked disease progression. However, these females still require less time to locate the “escape box” than males. Spatial learning and long-term memory at 10 months show pronounced deviations from healthy controls in both sexes (F(9, 843) = 4.805, females: 1 day—*p* = 0.0008, 2 day—*p* = 0.0008, 3 day—*p* = 0.0041, 4 day—*p* < 0.0001. males: 2 day—*p* = 0.0090, 3 day—*p* = 0.0101, 4 day—*p* < 0.0001. Tukey’s multiple comparisons test, Figure 5C) while simultaneously demonstrating measurable differences between male and female transgenic animals (1 day—*p* = 0.0034, 2 day—*p* < 0.0001, 3 day—*p* = 0.0011, Tukey’s multiple comparisons test, Figure 5C).

After 2.5 months, cognitive deficits persist. The latency to find the platform is statistically significantly increased in APP/Ps1 males compared to APP/Ps1 females (*p* = 0.0160, Kruskall-Wallis test, Figure 5D), and intergroup differences remain (*p* = 0.0062, *p* = 0.0107, Kruskall-Wallis test, Figure 5D). Furthermore, time spent in the target zone is substantially reduced in APP/Ps1 mice compared to control mice, indicating disease progression (*p* = 0.0072 (F), *p* = 0.0118(APP/Ps1 F × APP/Ps1 M), *p* < 0.0001 (M), Kruskall-Wallis test, Figure 5E). Long-term memory performance significantly differs between experimental and control animals compared to that at the 7.5-month time point. Notably, by 10 months of age, sex-dependent differences emerge within the APP/Ps1 group itself.

**Figure 6:**
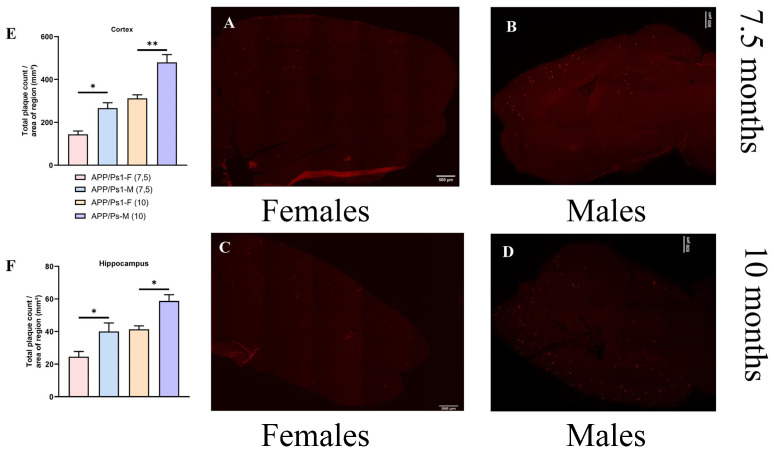
Histological analysis of amyloid deposits. Panoramic shooting in the TRITC fluorescence mode of a mouse brain slice was performed with a 10× lens in the NIS Elements AR version 4.6 application with frame stitching into a single composite image, 10% overlap and automatic post-processing, scale ruler—500 microns. (**A**) A snapshot of a 7.5-month-old female and (**B**) a snapshot of a 7.5-month-old male. (**C**) A snapshot of a 10-month-old female and (**D**) a snapshot of a 10-month-old male (**E**). The total number of amyloid accumulations in the cerebral cortex and (**F**) in hippocampus. * *p* < 0.05; ** *p* < 0.01.

The distribution of amyloid plaques shows a statistically significant lag in the number and area of aggregates in females compared to males at the same time points. The number of aggregates in 10-month-old females is at the same level as in 7.5-month-old males. The difference in the amount of amyloid deposits at 7.5 and 10 months has a statistical difference between females and males (F (3, 14) = 11.59, *p* = 0.0483 (F), *p* = 0.0341 (M), one-way ANOVA, Figure 6F). These differences are more pronounced in the cortex and increase over time (F (3, 14) = 23.19, *p* = 0.0143 (F), *p* = 0.0052 (M), one-way ANOVA, Figure 6E).

Sexual dimorphism in APP/PS1-associated pathology is manifested not merely in the difference in the total plaque burden, but in a qualitative difference in the nature of amyloidosis. In males, the pathological process is largely characterized by the accumulation of small plaques (<100 μm), regardless of the age of the study. We observed a predominance of the small plaque coefficient (Number of plaques/area of the zone) in males compared to that in females in both the hippocampal and cortical areas.

**Figure 7:**
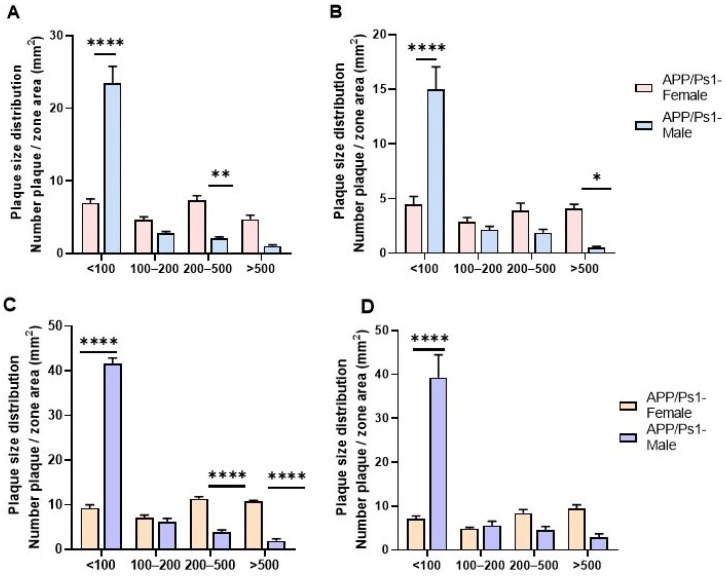
Histological analysis of amyloid deposits according to size. (**A**) The distribution of amyloid deposits according to size after 7.5 months in the cerebral cortex and (**B**) in the hippocampus. (**C**) The distribution of amyloid deposits according to size after 10 months in the cerebral cortex and (**D**) in the hippocampus. * *p* < 0.05; ** *p* < 0.01; **** *p* < 0.0001.

At 7.5 months of age, there was a highly significant difference in the cortex (*p* < 0.0001, F (3, 28) = 50.23, Sidak’s multiple comparisons test, Figure 7A), and similarly—in the hippocampus (*p* < 0.0001, F (3, 28) = 23.80, Sidak’s multiple comparisons test, Figure 7B). An analogous pattern of amyloid plaque formation was observed at 10 months of age (Cortex: F (3, 28) = 244.4, *p* < 0.0001, Figure 7C; Hippocampus: F (3, 28) = 20.24, *p* < 0.0001, Sidak’s multiple comparisons test, Figure 7D).

In females, despite a lower number of small plaques, a significantly greater number of mature, large plaques was observed. At 7.5 months, plaques sized 200–500 μm predominated in the cortex (*p* = 0.0043, Figure 7A), while in the hippocampus, very large plaques >500 μm were predominant (*p* = 0.0423, Figure 7B). By 10 months, the pattern of predominance of larger plaques in the cortex of females compared to that in males became more pronounced (plaques sized 200–500 μm and >500 μm had *p* < 0.0001, Sidak’s multiple comparisons test, Figure 7C), whereas in the hippocampus, the difference in the number of large plaques disappeared completely (Figure 7D).

This finding supports the hypothesis of a sexual dimorphism that extends beyond the total plaque burden to encompass the fundamental character of the amyloid pathology.

The number and localization of plaques directly correlate with the results of short-term and long-term memory testing (Figure S4). The observed “lag” in the onset of cognitive function deterioration in females compared to that in males may be directly dependent on the difference in the rate of amyloid accumulation.

**Figure 8:**
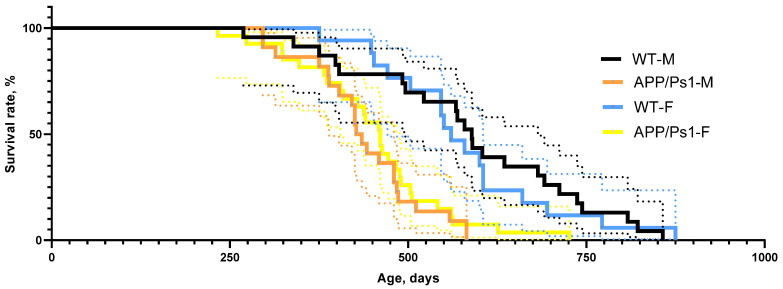
Survival curve.

No differences in the survival curve were found between female and male APP/Ps1 mice. The survival rate was statistically significantly different for both males and females when compared to that in control animals (*p* = 0.0006, *p* < 0.0001, Gehan-Breslow-Wilcoxon test, Figure 8).

## 4. Discussion

The present study provides compelling evidence for a profound sexual dimorphism in the phenotypic expression of Alzheimer’s disease-like pathology in the APPswe/PS1dE9/Blg transgenic mouse model. Our findings demonstrate that sex is a critical biological variable that not only influences the severity but also the temporal progression and behavioral manifestation of neurodegenerative changes, underscoring the necessity of including both sexes in preclinical AD research.

The study shows that the number of amyloid plaques, determined histologically, can serve as a structural biomarker of disease progression in transgenic models of Alzheimer’s disease. The observed sex differences in plaque accumulation and behavioral impairment highlight the importance of incorporating sex as a biological variable in biomarker discovery. Notably, the delayed plaque deposition and milder early cognitive decline in females resemble patterns reported in clinical neuroimaging studies, where women often maintain cognitive function despite comparable amyloid burden. This correspondence suggests that histomorphometric biomarkers in animal models may reflect in vivo neuroimaging signatures of resilience or compensation, strengthening their translational relevance.

Greater pathology correlates with a worse functional outcome—consistent with the concept of cognitive reserve and suggests that the female brain may have greater resistance or compensatory mechanisms against early amyloid toxicity [23,24].

The observed neuroprotection in females appears to be the result of synergy between several biological advantages.

For instance, 17β-estradiol exerts multiple protective effects on the brain [51]. Estrogen can modulate the activity of enzymes in the APP pathway, shifting APP processing toward the non-amyloidogenic pathway (alpha-secretase pathway), thereby reducing the production of Aβ peptide [52]. It is also involved in the formation of synapses, particularly in the hippocampus, which is directly linked to learning and memory. Furthermore, it participates in glucose metabolism and mitochondrial function in the brain, which may enhance neuronal resilience to stress.

The presence of two X chromosomes in females promotes greater stability in the expression of certain regulatory genes [53], such as KDM6A and KDM5C, which in turn help maintain cognitive function and neuronal plasticity [54]. These systems collectively create a “buffer” that may enhance the resilience of the female brain to the early stages of amyloid pathology.

Our results align with emerging clinical data indicating that women may tolerate higher levels of AD pathology before exhibiting clinical symptoms [44].

## 5. Conclusions

The key novelty of our study lies not in merely confirming sexual dimorphism in APP/PS1 mice, but in systematically identifying qualitatively distinct patterns of amyloid burden and their association with behavioral phenotypes under conditions of a restored neuroinflammatory response. Unlike previous studies [55], where sex was often considered a confounding variable, our design places biological sex at the forefront of the investigation. This approach enabled us to detect a temporal lag in pathology progression in females and establish histomorphometric parameters (plaque size distribution) as structural biomarkers significantly linked to cognitive deficits. These findings align with clinical observations of greater resilience in women to Alzheimer’s pathology [44] and advance translational disease modeling.

The conducted study established significant sex-dependent differences among the experimental groups. In the Novel Object Recognition test, WT females exhibited a higher RI than WT males, potentially indicating more pronounced exploratory activity. In contrast, APP/Ps1 animals of both sexes showed no significant preference for the novel object, consistent with the cognitive deficit characteristic of this model.

Analysis of the Y-maze test data revealed a complex picture of sex and genotype-dependent behavioral differences. Differences in exploratory behavior with sex-specific features were identified: APP/Ps1 mice of both sexes spent more time in the “old” arm of the maze, with this tendency being more pronounced in males. It is important to note that these behavioral patterns remained stable upon re-testing after 2.5 months, indicating the persistent nature of the observed changes.

The Barnes Maze test demonstrated more pronounced exploratory behavior in control mice compared to APP/Ps1 females (*p* < 0.01), whereas this difference was less marked in males (*p* < 0.05). Concurrently, APP/Ps1 males showed a lower learning ability in the Barnes Maze, manifested by an increased distance to find the escape box and a lack of learning progress. In contrast, APP/Ps1 females retained the capacity for learning at 7.5 months, albeit with reduced efficiency compared to controls. With age (by 10 months), transgenic animals of both sexes showed progression of cognitive impairments: worsening spatial learning and long-term memory, with these changes being more pronounced in males.

The analysis of amyloid plaque distribution revealed significant sexual dimorphism in the rate of their accumulation: females exhibited a significant lag in both the number (*p* = 0.0483 at 7.5 months and *p* = 0.0341 at 10 months) and area of aggregates compared to age-matched males. Particularly pronounced differences were observed in the cerebral cortex (*p* = 0.0143 and *p* = 0.0052, respectively), where the disparity between sexes progressively increased with age. Notably, the level of amyloid deposition in 10-month-old females corresponded to that in 7.5-month-old males.

This work identifies sex-dependent differences in both behavioral and histomorphometric biomarkers of Alzheimer’s disease progression in APPswe/PS1dE9/Blg mice. The results emphasize that amyloid plaque quantification can serve as a reliable morphological biomarker of a disease stage and functional decline. Integrating such measures with molecular and imaging-based approaches will enhance translational modeling and contribute to more precise, sex-aware strategies for early biomarker-driven diagnosis and therapy development.

Based on the obtained results, a number of proposals can be made for planning trials using this animal line. It is necessary to test drugs at several age points corresponding to pathology stages rather than chronological age. The inclusion criteria for the experiment should be revised to include not only sex-based [56] randomization but also additional baseline cognitive screening at study entry. When planning the experiment, it is essential to establish mixed-sex groups, for example: “males on active therapy”, “males on placebo”, “females on active therapy”, and “females on placebo”.

## Data Availability

The raw data supporting the conclusions of this article will be made available by the authors on request.

## Supplementary Materials

The following supporting information can be downloaded at: https://www.mdpi.com/article/10.3390/brainsci15111237/s1. Figure S1: Experimental Design. At the ages of 7.5 and 10 months, behavioral tests were performed for 2 weeks with the mice “resting” after each test; Figure S2. Alterations in emotionality in APP/PS1 mice at the age of 7.5 months; Figure S3: Alterations in emotionality in APP/PS1 mice at the age of 10 months; Figure S4: Data from the correlation analysis of the dependence of amyloid deposits accumulation and behavioral testing.

## Abbreviations

The following abbreviations are used in this manuscript:

AD	Alzheimer’s disease.
APP/Ps1	APPswe/PS1dE9/Blg
Aβ	Amyloid beta
PET	Positron emission tomography
MRI	Magnetic Resonance Imaging
DI	Discrimination Index
RI	Recognition Index
