# Supplementary material for: Sex-Dependent Phenotypic and Histomorphometric Biomarkers in the APPswe/PS1dE9/Blg Mouse Model of Alzheimer’s Disease
Source: Brain Sci. 2025 Nov 18;15(11):1237. doi: 10.3390/brainsci15111237 (PMC12651399; doi:10.3390/brainsci15111237)
Supplement: Supplementary file 1 [file brainsci-15-01237-s001.zip › brainsci-3974258-supplementary.pdf]

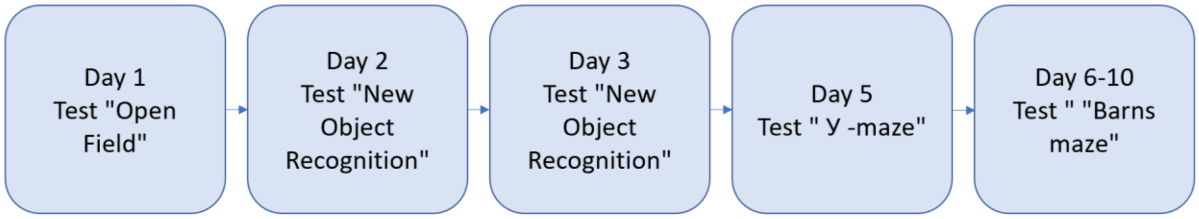

Figure S1. Experimental Design. At the ages of 7.5 and 10 months, behavioral tests were performed for 2 weeks with the mice "resting" after each test.

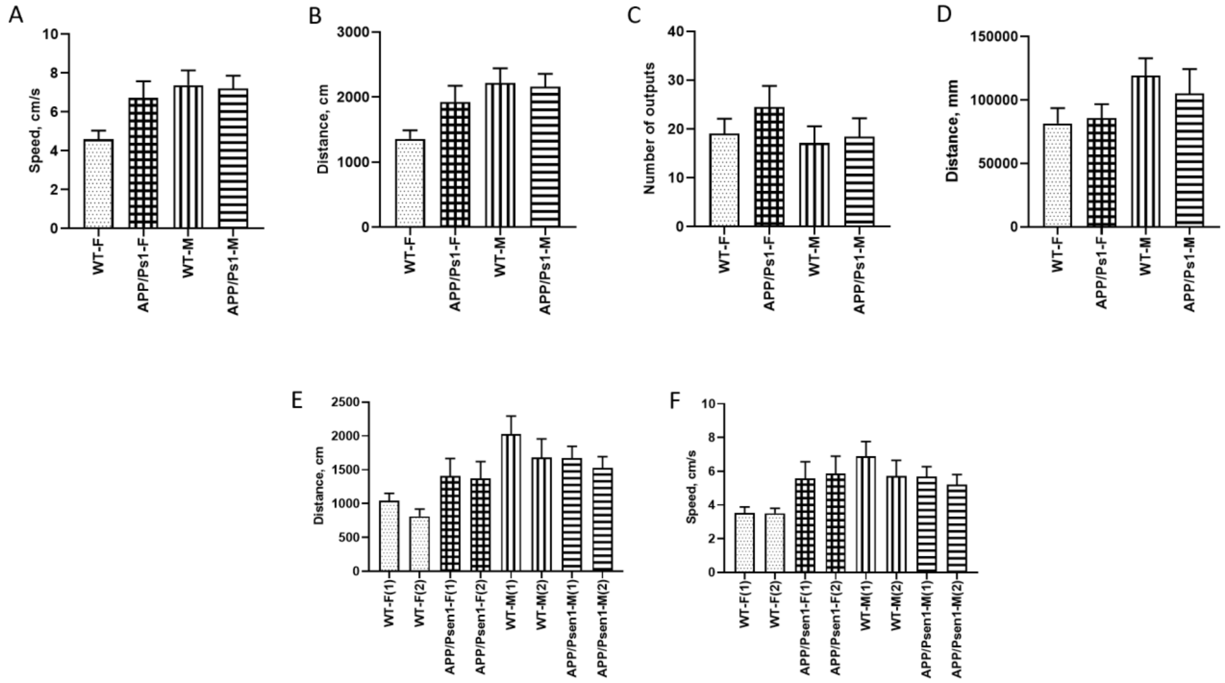

Figure S2. Alterations in emotionality in APP/PS1 mice at the age of 7.5 months. In the "Open Field" test, there were no significant differences between the groups in general locomotor indicators, such as speed (A) and distance (B), as well as (C) the total number of sectors crossed. Overall locomotor performance I have no statistical differences in the "New Object Recognition" test (D), as well as in the "Y-maze" test in distance (E) and speed (F). Statistical analyses were conducted using two-way and one-way ANOVA.

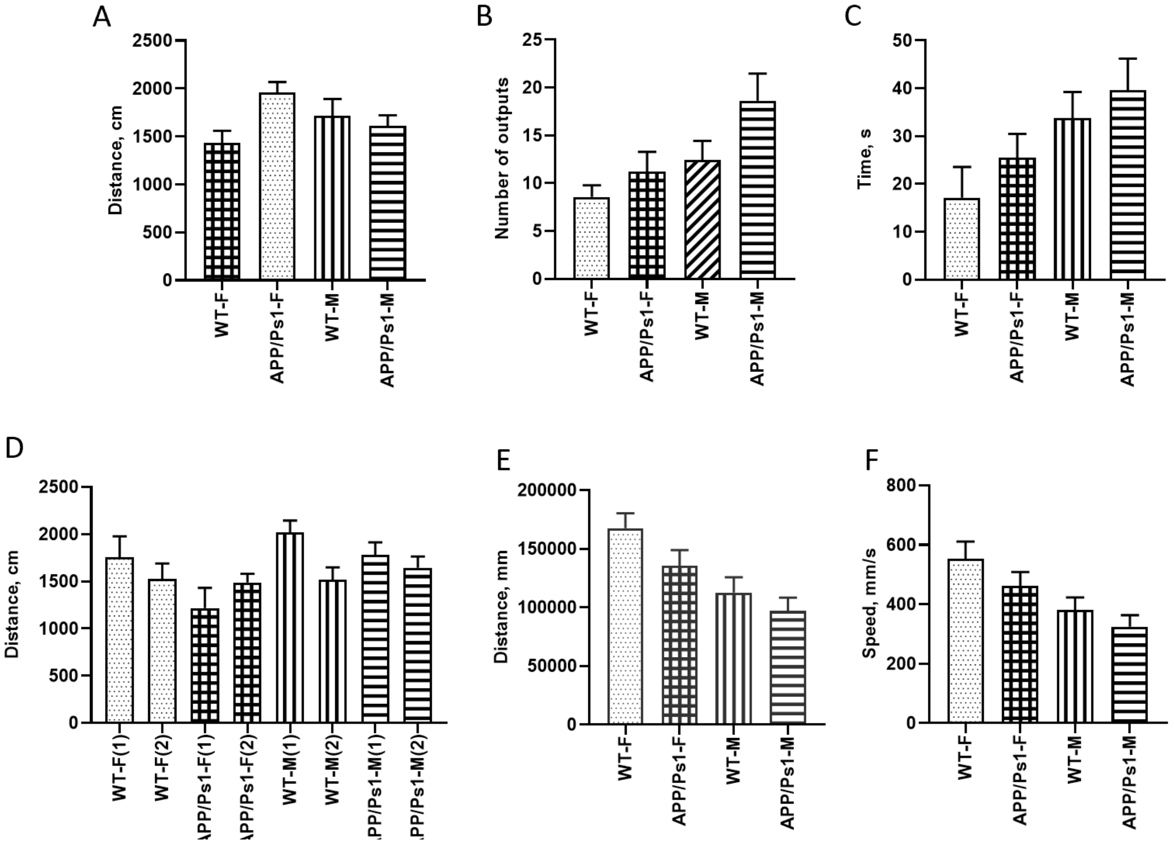

Figure S3. Alterations in emotionality in APP/PS1 mice at the age of 10 months. In the "Open Field" test, there were no significant differences between the groups in general locomotor indicators, such as distance (A), as well as (B) the total number of sectors crossed, and time spent in different sectors(C). Overall locomotor performance I have no statistical differences in the "New Object Recognition" test (D), as well as in the "Y-maze" test in distance (E) and speed (F). Statistical analyses were conducted using two-way and one-way ANOVA.

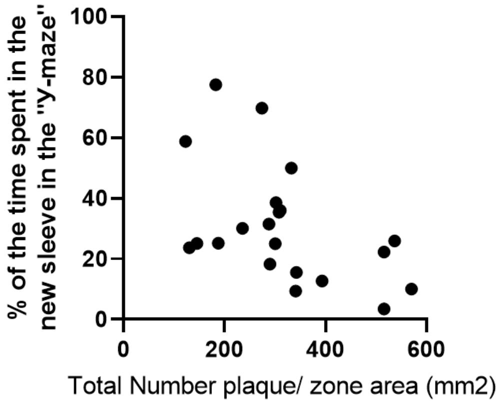

Figure S4. Data from the correlation analysis of the dependence of amyloid deposits accumulation and behavioral testing. When conducting a correlation analysis between the accumulation of amyloid plaques and behavioral testing, we found a direct pattern. With an increase in the number of amyloid deposits, the time spent by mice in the new arm in the "Y-maze" test also decreases.

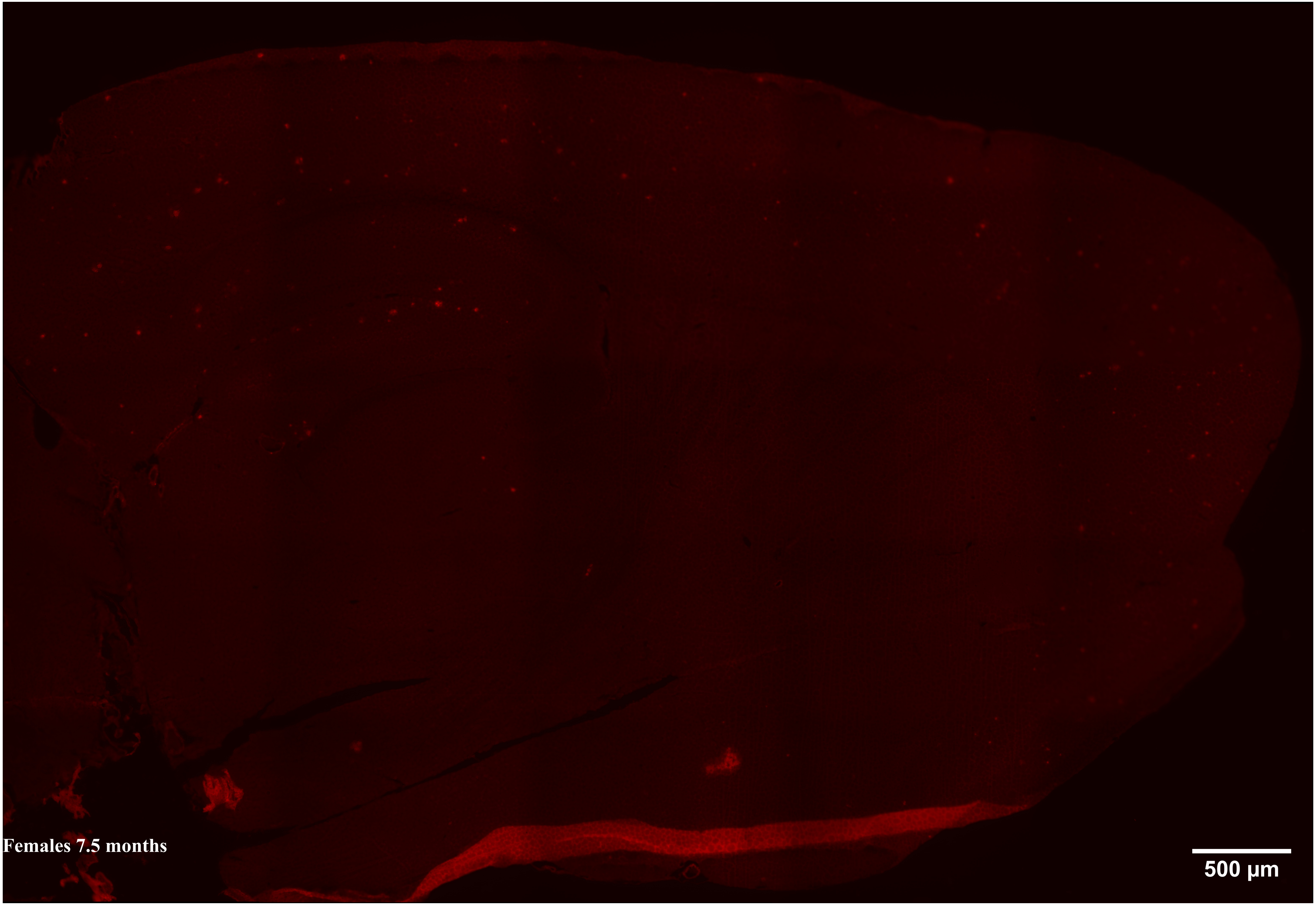

Females 7.5 months
500 µm

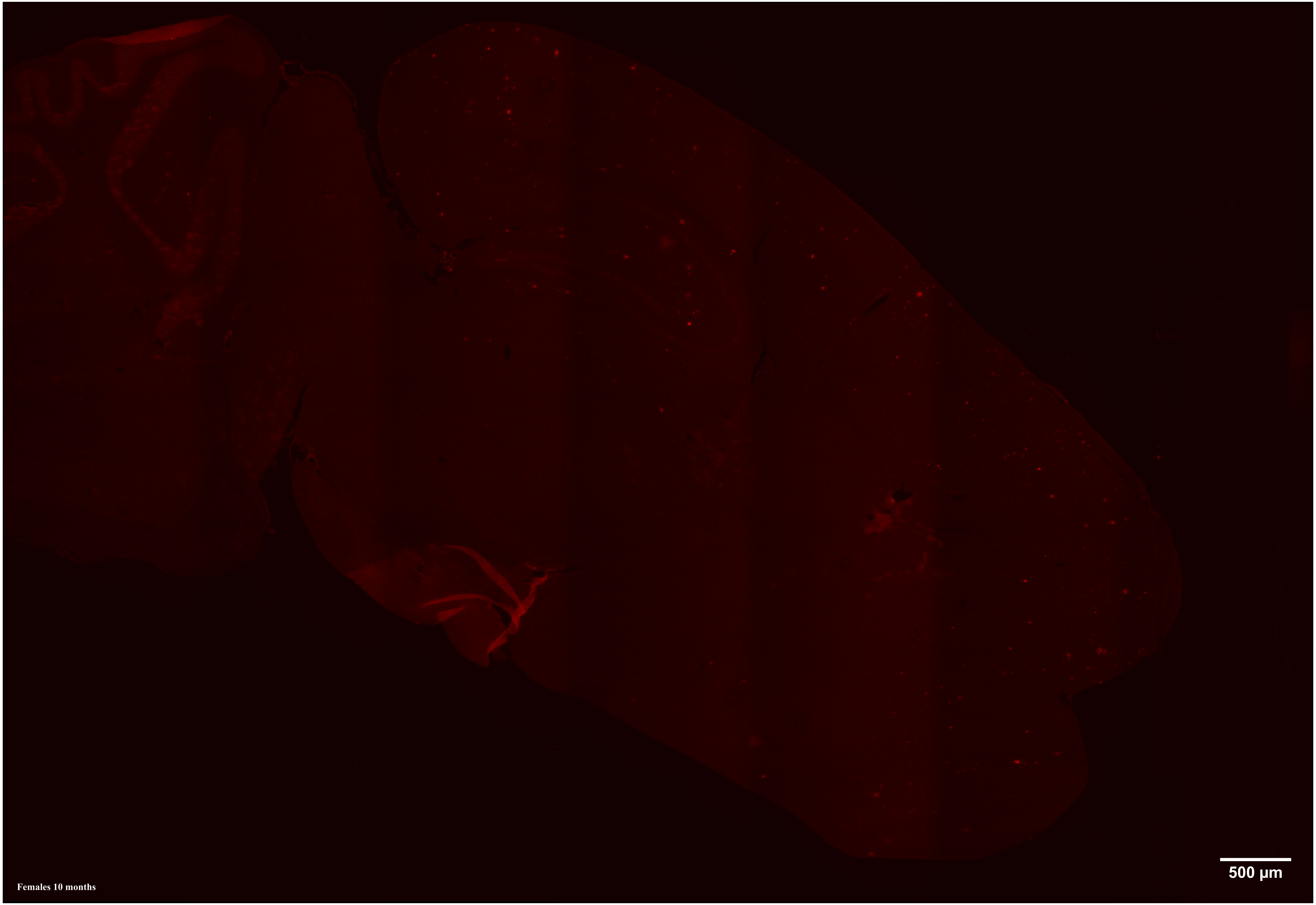

Females 10 months

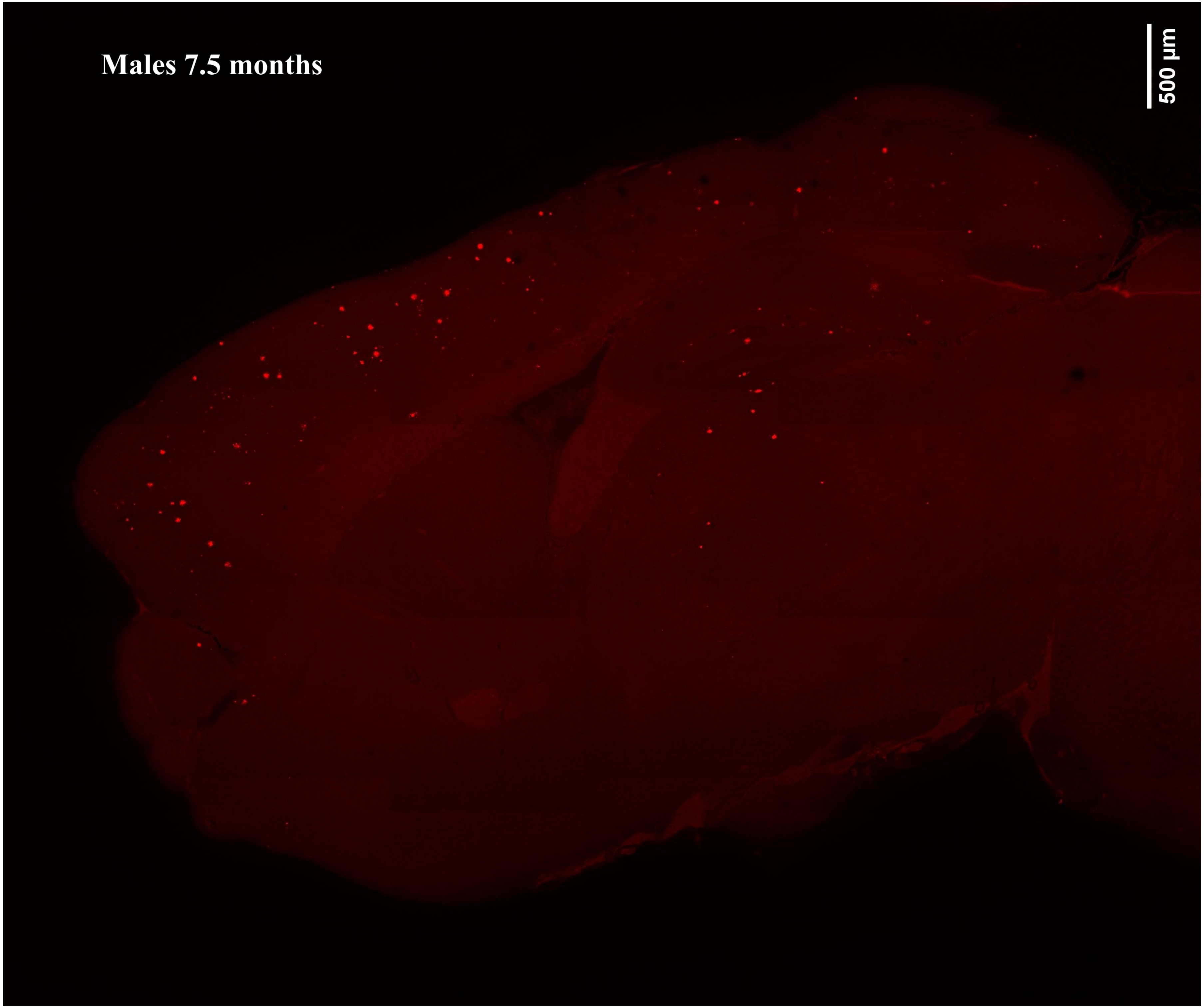
Males 7.5 months
500 µm

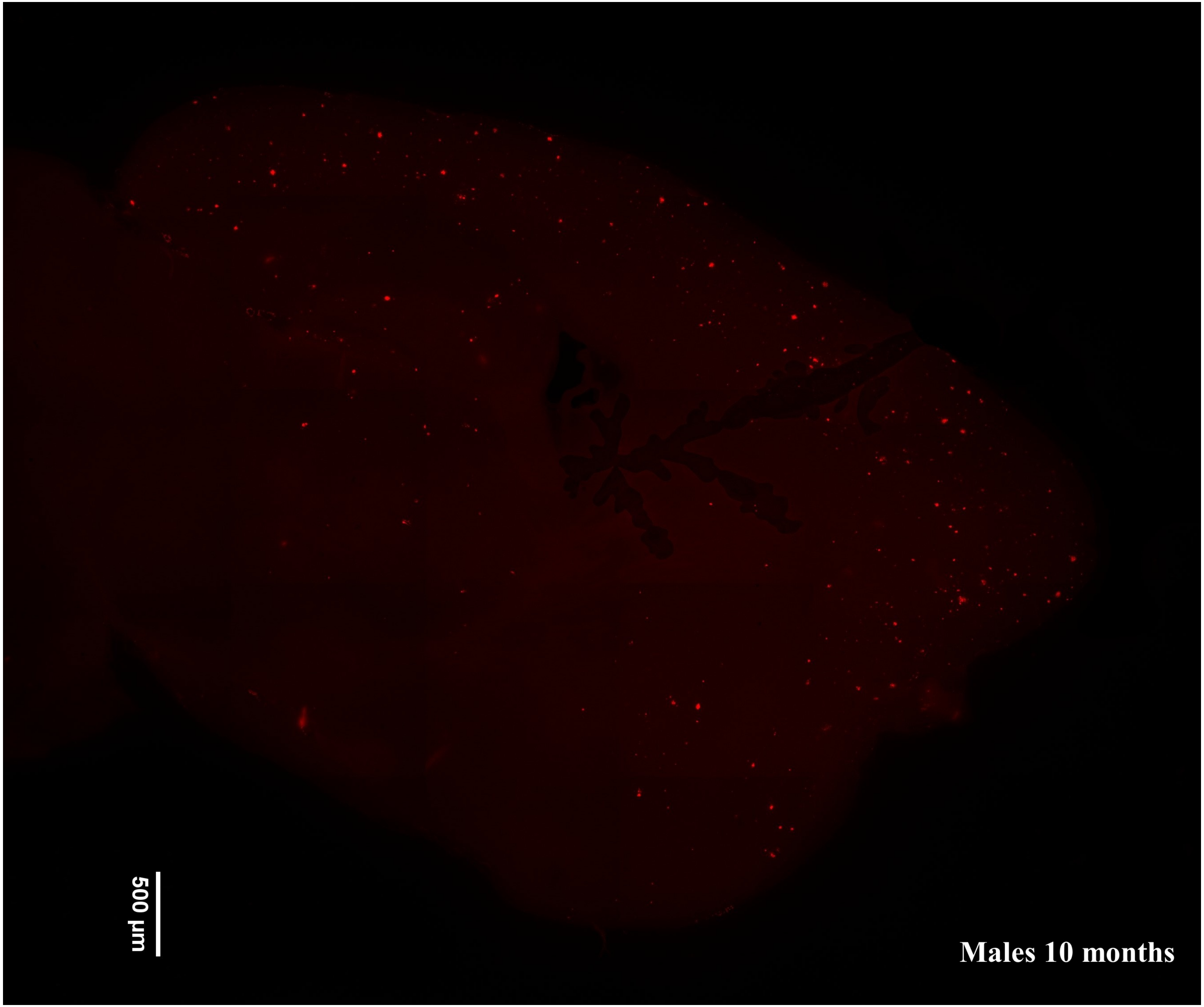
500 µm
Males 10 months
